# A Novel Galantamine-Curcumin Hybrid as a Potential Multi-Target Agent against Neurodegenerative Disorders

**DOI:** 10.3390/molecules26071865

**Published:** 2021-03-25

**Authors:** Rumyana Simeonova, Dimitrina Zheleva, Iva Valkova, Georgi Stavrakov, Irena Philipova, Mariyana Atanasova, Irini Doytchinova

**Affiliations:** 1 Faculty of Pharmacy, Medical University of Sofia, 1000 Sofia, Bulgaria; rsimeonova@pharmfac.mu-sofia.bg (R.S.); dzheleva@pharmfac.mu-sofia.bg (D.Z.); ivalkova@pharmfac.mu-sofia.bg (I.V.); stavrakov@pharmfac.mu-sofia.bg (G.S.); matanasova@pharmfac.mu-sofia.bg (M.A.); 2 Institute of Organic Chemistry with Centre of Phytochemistry, Bulgarian Academy of Sciences, 1113 Sofia, Bulgaria; irena@orgchm.bas.bg

**Keywords:** galantamine, curcumin, in vivo AChE inhibition, acute toxicity in mice, BBB permeability by PAMPA, antioxidant activity, malondialdehide levels, glutathione levels, brain homogenate, complete blood count, biochemical serum parameters, DPPH, ABTS, FRAP, LPO inhibition

## Abstract

The acetylcholinesterase (AChE) inhibitors are the main drugs for symptomatic treatment of neurodegenerative disorders like Alzheimer’s disease. A recently designed, synthesized and tested hybrid compound between the AChE inhibitor galantamine (GAL) and the antioxidant polyphenol curcumin (CU) showed high AChE inhibition in vitro. Here, we describe tests for acute and short-term toxicity in mice as well as antioxidant tests on brain homogenates measured the levels of malondialdehide (MDA) and glutathione (GSH) and in vitro DPPH, ABTS, FRAP and LPO inhibition assays. Hematological and serum biochemical analyses were also performed. In the acute toxicity tests, the novel AChE inhibitor given orally in mice showed LD_50_ of 49 mg/kg. The short-term administration of 2.5 and 5 mg/kg did not show toxicity. In the ex vivo tests, the GAL-CU hybrid performed better than GAL and CU themselves; in a dose of 5 mg/kg, it demonstrates 25% reduction in AChE activity, as well as a 28% and 73% increase in the levels of MDA and GSH, respectively. No significant changes in blood biochemical data were observed. The antioxidant activity of **4b** measured ex vivo was proven in the in vitro tests. In the ABTS assay, **4b** showed radical scavenging activity 10 times higher than the positive control butylhydroxy toluol (BHT). The GAL-CU hybrid is a novel non-toxic AChE inhibitor with high antioxidant activity which makes it a prospective multitarget drug candidate for treatment of neurodegenerative disorders.

## 1. Introduction

The neurodegenerative disorders like Alzheimer’s and Parkinson’s diseases have increased exponentially each decade and during the next few decades will overtake cancer and become the second leading cause of death after cardiovascular diseases [1]. The successful treatment of these disorders requires reliable tests and biomarkers for early diagnosis, identification of translational drug targets and fast discovery and development of multitarget drugs and therapeutic strategies to tackle the complex pathogenesis of the neurodegeneration. 

**Figure 1:**
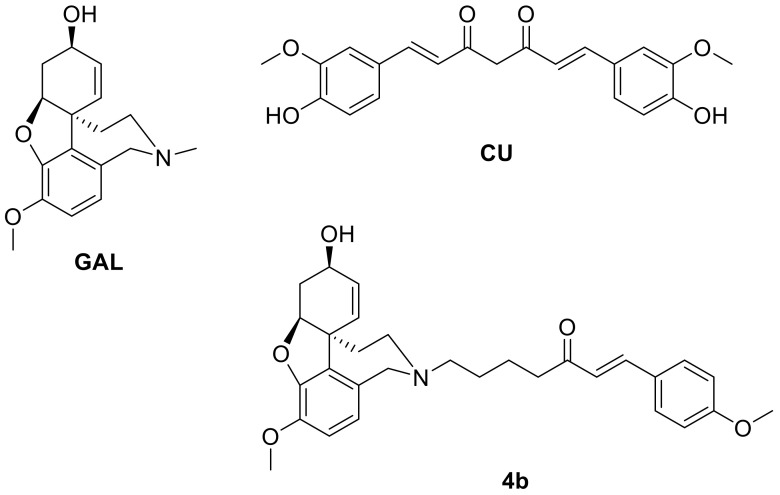
Structures of galantamine (GAL), curcumin (CU) and their hybrid **4b**.

In the last 20 years, the main drugs used to cope with some of the symptoms of Alzheimer’s disease (AD) are inhibitors of the enzyme acetylcholinesterase (AChE). The reversible AChE blockage increases the levels of acetylcholine (ACh)—the main neurotransmitter associated with the cognitive and motor brain functions. Galantamine (GAL) (Figure 1) is among the few AChE inhibitors approved for treatment of AD [2]. Additionally, GAL is an allosteric modulator of nicotinic and muscarinic ACh receptors [3,4], increases the phagocytosis of amyloid β (Aβ) peptide in rat microglia [5] and exhibits a moderate scavenging effect in antioxidant studies [6].

The oxidative stress hypothesis in AD dates back to the late 1990s [7,8]. According to this hypothesis, the mammal’s brain is very sensitive to oxidative stress because of the abundant presence of polyunsaturated fatty acids and transition metals like iron, copper and zinc [9] and the relative shortage of antioxidant ability to detoxify the free radicals [10]. Due to the oxidative stress, the cholinergic neurons undergo a degeneration leading to impairments in cognition and memory. One of the strategies for prevention and treatment of Alzheimer’s disease is focused on the suppression of oxidative damage [7,8]. Most of the clinical trials report associations between antioxidant use and better cognitive functions [11,12,13,14,15]. 

Curcumin (CU) (Figure 1) is a natural polyphenol with a powerful antioxidant activity [16] and ability to reduce oxidative stress and amyloid pathology in transgenic mice [17]. Additionally, it was found that CU binds to Aβ oligomers and fibrils and inhibits the β-sheet formation [18]. We simulated the primary nucleation of Aβ peptide by molecular dynamics and showed that CU molecules inhibit the process by intercalating among the Aβ chains [19]. Moreover, CU is able to disintegrate preformed Aβ fibrils [18], reduce insoluble Aβ deposits and amyloid plaques [20]. Curcumin has already been used as a supplement for the treatment of various medical conditions such as: hair loss [21], inflammations [22], microbial and parasitic infections [23,24], different types of cancers [25,26].

Recently, we designed a combinatorial library of GAL-CU hybrids, screened it for optimal ADME properties and blood–brain permeability and docked on AChE [19]. The 14 best performing hybrids were synthesized and tested for neurotoxicity and AChE inhibition in vitro. Five of them showed less toxicity than GAL and CU and AChE inhibition between 41 and 186 times higher than GAL. Here, we describe the in vivo evaluation of acute and short-term toxicity and the ex vivo and in vitro antioxidant properties of the best performing inhibitor—compound **4b**. As the present study is a continuation of our previous research [19] on this particular compound, we prefer to keep the same compound ID. As positive controls, GAL and CU are used in the study.

## 2. Materials and Methods

### 2.1. Materials

In the present study, were used the following materials: galantamine HBr (Galen-N Ltd., Sofia, Bulgaria, Mw = 368.3 g/mol, purity > 98%), curcumin (BioXtract, Les Isnes, Belgium, Mw = 368.4 g/mol, purity > 98%), bovine serum albumin (Sigma Aldrich, Taufkirchen, Germany, Mw ≈ 66 kD, purity > 96%), thiobarbituric acid (Sigma Aldrich, Mw = 144.15 g/mol, purity > 98%), trichloroacetic acid (Sigma Aldrich, Mw = 163.39 g/mol, purity > 99%), acetylthiocholineiodide (Sigma Aldrich, Mw = 289.18, purity > 98%), 2,2- dinitro-5,5 dithiodibenzoic acid (DTNB) (Sigma Aldrich, Mw = 396.35, purity > 98%), ethylenediaminetetraacetic acid (EDTA) (Sigma Aldrich, Mw = 292.24, purity > 99%). 2,2′-diphenyl-1-picrylhydrazyl (DPPH), 2,2′-azinobis- (3-ethylbenzothia-zine-6-sulfonic acid) (ABTS), 6-hydroxy-2,5,7,8-tetra- methylchroman-2-carboxylic acid (Trolox), 2,4,6-tripyridyl-s-triazine (TPTZ), FeCl_3_ × 6H_2_O, sodium acetate, potassium persulfate and butylhydroxy toluol (BHT) were purchased from Sigma-Aldrich. All other chemicals including the solvents were of analytical grade. 

The synthesis of **4b** and detailed analytical data are already reported in the context of the synthesis of a series of GAL-CU hybrids [19]. A contribution of the herein reported synthetic procedures is the scale-up and optimization of the protocols.

**Scheme 1:**
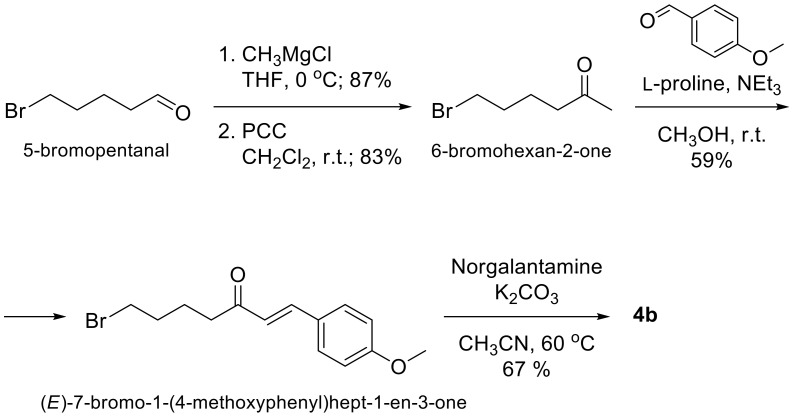
Synthesis of **4b**.

A stirred solution of 5-bromopentanal (1.038 g, 6.29 mmol) in dry THF (25 mL) under argon atmosphere was treated dropwise at 0 °C with CH_3_MgCl (3 M in THF, 4.2 mL, 12.58 mmol) (Scheme 1). The reaction was stirred for 30 min at r.t., cooled down to 0 °C and quenched with 10 mL sat.aq.NH_4_Cl. The resulting mixture was extracted (ether), dried (MgSO_4_), filtered, concentrated and purified by flash column chromatography to give 6-bromohexan-2-ol in 87% yield. A suspension of 6-bromohexan-2-ol (0.782 g, 4.32 mmol), PCC (1.862 g, 8.64 mmol) and Celite (0.500 g) in CH_2_Cl_2_ (25 mL) was stirred for 18 h at r.t. The mixture was diluted with petroleum ether (75 mL), filtered through a pad of Celite, concentrated and subjected to flash column chromatography to give 6-bromohexan-2-one in 83% yield.

A solution of 6-bromohexan-2-one (0.615 g, 3.435 mmol), *p*-methoxybenzaldehyde (0.562 g, 4.122 mmol), l-proline (0.06 g, 0.515 mmol) and NEt_3_ (0.104 g, 1.031 mmol) in methanol (7 mL) was stirred at r.t. for 5 days. The mixture was concentrated and directly subjected to flash column chromatography to give (*E*)-7-bromo-1-(4-methoxyphenyl)hept -1-*en*-3-one in 59% yield. The latter bromide (0.248 g, 0.834 mmol) and anhydrous K_2_CO_3_ (0.314 g, 2.276 mmol) were added to a solution of Norgalantamine (0.207 g, 0.759 mmol) in anhydrous acetonitrile (20 mL) under argon atmosphere. After stirring for 24 h at 60 °C, the mixture was filtered through a pad of Celite, concentrated and purified by flash column chromatography to give 0.249 g (67%) of the desired product **4b**.

### 2.2. Animals

Male and female ICR mice (6 weeks old, 25–35 g) obtained from the National Breeding Center, Sofia, Bulgaria, were used in the experiments. As a more sensitive sex [27], 12 females were used in the acute toxicity test and 30 males in the short-term toxicity test. Mice were housed in Plexiglas cages (6 per cage) in a 12/12 light/dark cycle, under standard laboratory conditions (ambient temperature 20 ± 2 °C and humidity 72 ± 4%) with free access to water and standard pelleted food No. 53-3, produced in accordance with ISO9001:2008. Prior to the start of the experiment, the mice were acclimatized under vivarium conditions for seven days and their health was monitored daily by a veterinarian. The vivarium (registration certificate No. 15320139/01.08.2007) was inspected by the Executive Agency for Medicines in order to verify the conditions for keeping laboratory animals (No. A-16-0532/14.10.2016). All experiments strictly followed the principles set out in the European Convention for the Protection of Vertebrate Animals Used for Experimental and Other Scientific Purposes (ETS 123) (Council of Europe, 1991). Efforts have been made to minimize animal suffering. The Animal Care Ethic Committee approved the study protocol and Ethic clearance (No. 273 from 02/06/2020) was issued for the study by the Bulgarian Agency for Food Safety.

### 2.3. Acute Toxicity Protocol

Acute toxicity testing was performed on female mice after oral (po) administration of **4b** using a simplified Lorke’s method [28]. Three animals per dose were used, starting with a dose of 10 mg/kg and gradually increasing it every 3 h until the first lethal case. Compound **4b** was solubilized with Tween 80 (0.1%) in distilled water and administered orally in a volume of 0.1 mL/10 g.

The LD_50_ is calculated using the following equation:

(1) $$LD_{50}=\sqrt{\left(D_{0}\times D_{100}\right)}$$

where *D*_0_ is the highest dose that does not cause mortality and *D*_100_ is the lowest dose that results in death.

Surviving animals were observed for 24 h and then for up to 14 days. On day 14, they were euthanized after anesthesia with ketamine/xylazine (80/10 mg/kg, i.p.) and an examination of the internal organs for possible macroscopic abnormalities (organ color, consistency, neoplasms, etc.) was made.

### 2.4. Short-Term Toxicity Protocol

The short-term toxicity test was performed on male ICR mice at the same age of 6 weeks and weighing between 25 and 35 g. Three drugs (GAL, CU and **4b**) were administered daily for 14 days orally with a dosing needle at approximately the same time of day, 11:00 a.m. Based on the LD_50_ value ≈ 50 mg/kg, derived in the acute toxicity test, two doses of 2.5 mg/kg and 5 mg/kg (1/20 and 1/10 of the LD_50_) were selected for multiple administration of **4b**. The animals were divided into 5 experimental groups of 6 mice (*n* = 6). Group 1 was the control group treated with physiological saline via oral gavage (0.1 mL/10 g bw); group 2 was treated orally with the positive control GAL in dose of 3 mg/kg dissolved in distilled water [29]; group 3 was treated orally with the second positive control CU at a dose of 25 mg/kg [30]; group 4 was treated orally with **4b** at a dose of 2.5 mg/kg (1/20 LD_50_); group 5 was treated orally with **4b** at a dose of 5 mg/kg (1/10 LD_50_). 

Animals were observed daily for behavioral changes and signs of toxicity.

On day 15, after 14 days of treatment, the animals were anesthetized with ketamine/xylazine (80 mg/10 kg ip) and decapitated. Blood was taken in vacutainers for complete blood count and biochemistry measurement. The brains were dissected, measured and prepared for the assessment of AChE inhibition, malondialdehide (MDA) and glutathione (GSH) levels. Protein content of brain homogenates was measured by the method of Lowry [31] using bovine serum albumin as a standard.

### 2.5. Measurement of Blood–Brain Barrier (BBB) Permeability

The ability of GAL, CU and **4b** to cross the BBB by passive diffusion was tested in vitro by Parallel Artificial Membrane Permeability Assay (PAMPA) on Permeability Analyser (pION Inc., Billerica, MA, USA). According to the manufacturer’s protocol, the tested compounds and the controls were prepared as 10 mM stock solutions in DMSO. Prisma HT buffer with pH 7.4 was used further for preparing the donor phase. Lipid formulation BBB-1 was used as an artificial membrane; no stirring was applied. The permeation was measured spectrophotometrically at a wavelength of 250–500 nm for 4 h at temperature 25 °C. The permeability was presented as −log*P_e_*, where *P_e_* is the permeability coefficient (10^−6^ cm/s). Compounds with −log*P_e_* below 5 (*P_e_* > 10^−5^ cm/s) are considered as highly permeable, those with −log*P_e_* between 5 and 6 (10^−6^ cm/s < *P_e_* < 10^−5^ cm/s) as medium permeable and those with −log*P_e_* above 6 (*P_e_* < 10^−6^ cm/s) as low permeable [32]. Theophylline, progesterone, and propranolol were used as controls.

### 2.6. Measurement of Acetylcholinesterase (AChE) Inhibition in Brain Homogenate

Brains were homogenized with 0.1 M phosphate buffer, pH 7.4. Aliquots of brain homogenates from different groups were used to measure AChE activity for 10 min by the method of Ellman [33]. AChE activity was calculated and expressed as nmol/min/mg protein using a molar absorption coefficient of 13,600 M^−1^ cm^−1^.

### 2.7. Measurement of Malondialdehyde (MDA) Levels in Brain Homogenate

Brains were homogenized with 0.1 M phosphate buffer and EDTA, pH 7.4. Aliquots of the homogenates were heated for 20 min on a water bath (100 °C) with thiobarbituric acid. The amount of thiobarbituric acid-formed reactive species (TBARS) (expressed as MDA equivalents) was measured spectrophotometrically by the method of Deby and Goutier [34] at a wavelength of 535 nm. The concentration of MDA was calculated using a molar absorption coefficient of 1.56 × 10^5^ M^−1^ cm^−1^ and expressed in nmol/g tissue.

### 2.8. Measurement of Glutathione (GSH) Levels in Brain Homogenate

GSH was evaluated by measuring non-protein sulfhydryls after trichloroacetic acid (TCA) protein precipitation by the method described by Bump et al. [35]. Brains were homogenized in 5% TCA (1:10) and centrifuged for 20 min at 4000× *g*. The reaction mixture contained 0.05 mL supernatant, 3 mL 0.05 M phosphate buffer (pH = 8), and 0.02 mL DTNB reagent. Absorption was determined at a wavelength of 412 nm and the results were expressed as nmol/g tissue.

### 2.9. Measurement of Hematological and Serum Biochemical Data

Whole blood was analyzed by a semi-automated hematological analyzer BC-2800 Vet, (Mindray, Shenzhen, China) according to the manufacturer’s instructions. The count of leukocytes (WBC), erythrocytes (RBC, Er), platelets (PLT), amount of hemoglobin (Hb), hematocrit (Ht), as well as the differential count of leukocytes were measured. The biochemical serum data as blood sugar (GLU), cholesterol (CHOL), triglycerides (TRIG), urea (UREA), creatinine (CREAT), total protein (TP), albumin (ALB), total bilirubin -Bil), direct bilirubin (D-Bil), calcium (Ca), uric acid (Uric acid), as well as the activity of the enzymes aspartate aminotransferase (ASAT), alanine aminotransferase (ALAT), alkaline phosphatase (ALP), gamma glutamyl transferase (GGT) and amylase (AMYL) were measured using automated biochemistry analyzer kits (BS-120, Mindray), following the manufacturer’s instructions.

### 2.10. Measurement of 2-Diphenyl-1-picrylhydrazyl (DPPH) Radical Scavenging Activity

Free radical scavenging activity was measured by DPPH method [36]. An amount of 1 mL of each compound (1 mM) in MeOH was added to 1 mL methanol solution of DPPH (0.05 mM). The absorbance was measured at 517 nm after 30 min using a Shimatzu 1203 UV–VIS spectrophotometer (Kyoto, Japan). The results were expressed as μM Trolox equivalent (TE). The antioxidant butylated hydroxytoluene (BHT) (1 mM in MeOH) was used as a positive control.

### 2.11. Measurement of 2,2′-Azino-bis(3-ethylbenzothiazoline-6-sulfonic acid (ABTS) Radical Scavenging Activity

For ABTS assay, the procedure followed the method of Grochowski [36] with some modifications. The stock solutions included 7 mM ABTS solution and 2.4 mM potassium persulfate solution. The tested solution was prepared by mixing the two stock solutions in equal quantities and allowing them to react for 14 h at room temperature in the dark. The solution was then diluted by mixing 2 mL ABTS solution with 50 mL methanol to obtain an absorbance of 0.705 ± 0.05 units at 734 nm using a Shimatzu 1203 UV–VIS spectrophotometer. A fresh ABTS solution was prepared for each assay; 0.1 mM of each compound (0.5 mL) was allowed to react with 1.5 mL of the ABTS solution, and the absorbance was taken at 734 nm after 5 min. The results were expressed as Trolox equivalent (TE). BHT (1 mM in MeOH) was used as a positive control.

### 2.12. Measurement of Ferric Reducing Antioxidant Power (FRAP)

The FRAP assay was done according to the method described by Grochowski [36] with some modifications. The stock solutions included 300 mM acetate buffer pH 3.6, 10 mM TPTZ solution in 40 mM HCl, and 20 mM FeCl_3_·6H_2_O solution. The fresh tested solution was prepared by mixing 25 mL acetate buffer, 2.5 mL TPTZ solution, and 2.5 mL FeCl_3_·6H_2_O solution and then warmed at 37 °C before use. A volume of 150 µL of 1 mM solution of compounds in MeOH was allowed to react with 2.85 mL of the FRAP solution for 30 min in dark conditions. Readings of the colored product (ferrous tripyridyltriazine complex) were then taken at 593 nm. Results were expressed as Trolox equivalent (TE). BHT (1 mM in MeOH) was used as a positive control.

### 2.13. Measurement of Inhibition of Lipid Peroxidation

Determination of the inhibition of lipid peroxidation (LPO) in linoleic acid system was measured by ferric thiocyanate assay (FTC) [37]. The tested solution, containing 0.6 mL of compound (10 mM in MeOH), 0.6 mL of linoleic acid emulsions (25 mg/mL in 99% ethanol), and 1.2 mL of 50 mM phosphate buffer (pH 7.4), was incubated in the dark at 40 °C. A 0.1 mL aliquot of it was then added to 3 mL of 70% (*v*/*v*) ethanol and 0.2 mL of 30% (*w*/*v*) ammonium thiocyanate. Precisely 3 min after the addition of 0.2 mL of 20 mM ferrous chloride in 3.5% (*v*/*v*) hydrochloric acid to the reaction mixture, the absorbance of the resulting red color was measured at 500 nm. Aliquots were assayed every 24 h for five days. BHT (10 mM in MeOH) was used as a positive control. MeOH was used as a blank.

### 2.14. Statistical Analysis

The MEDCALC statistical program (MedCalc Software, v. 12.3) was used to analyze the in vivo data. Results are expressed as mean ± SD for six animals in each group. The significance of the data was assessed by a nonparametric Mann–Whitney U test. Values of *p* ≤ 0.05 are considered as statistically significant.

## 3. Results

### 3.1. Acute Toxicity in Mice

The median lethal dose (LD_50_) of **4b** in mice was 49 mg/kg. No serious toxic effects or mortality were observed in mice at doses of 10 and 20 mg/kg. At dose of 40 mg/kg, tachypnea and mild tremor for up to 2 h were observed with no other visible signs of toxicity. At dose of 60 mg/kg, one fatal outcome was observed 3 h post-dosing with accelerated breathing, piloerection, mild tremor and seizures. Fourteen days later, the animals were euthanized and a macroscopic examination of the internal organs was performed. No changes in the size, color and consistency of the lungs, liver, heart, kidneys, stomach, spleen, and intestine were observed. No abnormalities in the morphology of the gonads and brain were detected.

### 3.2. Short-Term Toxicity in Mice

The short-term toxicity of **4b** was assessed by daily administration of 2.5 mg/kg (1/20 of LD_50_) and 5 mg/kg (1/10 of LD_50_) for 14 days. As positive controls, GAL in dose of 3 mg/kg (1/10 of LD_50_) and CU in dose of 25 mg/kg (1/10 of LD_50_) were used. A control (placebo) group also was included in the test. Animals were examined daily and no behavioral changes and signs of toxicity were observed. On day 15, the animals were anesthetized and decapitated. Blood from each group was collected for hematological and serum biochemical analyses. Brains from each group were collected and prepared for measurements of AChE inhibition, MDA and GSH levels. 

### 3.3. Blood–Brain Barrier (BBB) Permeability

The BBB permeability of the tested compounds was measured in vitro by PAMPA test. Values are given as −log*P_e_*, where *P_e_* is the permeability coefficient (10^−6^ cm/s). Compounds with −log*P_e_* above 6 are considered as low BBB permeable, those with −log*P_e_* between 5 and 6 as medium permeable, and those with −log*P_e_* below 5 as highly permeable. The results from the PAMPA test were as follows: −log*P_e_* (GAL) = 5.448, −log*P_e_* (CU) = 5.075 and −log*P_e_* (**4b**) = 4.593. Accordingly, GAL and CU have intermediate BBB permeability, while **4b** is highly BBB permeable. 

### 3.4. AChE Inhibition in Mice Brain Homogenate

**Figure 2:**
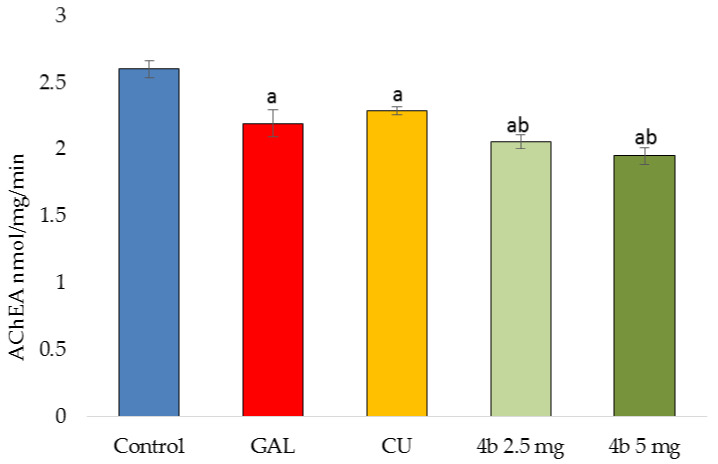
Acetylcholinesterase (AChE) activity measured for 10 min in brain homogenates derived from mice treated 14 days by GAL, CU, **4b** in two doses (2.5 mg/kg and 5 mg/kg) or placebo (control group). ^a^*p* < 0.05 vs. control ^b^*p* < 0.05 vs. CU.

After 14 days of treatment of mice by GAL, CU, **4b** or placebo, the AChE activity was measured for 10 min by the Ellman’s method. The results are given in Figure 2. CU caused a mild decrease in AChE activity, followed by GAL and **4b**. The dose of 5 mg/kg causes 25% inhibition of enzyme activity compared to the control group. 

### 3.5. Antioxidant Activity in Mice Brain Homogenate

**Figure 3:**
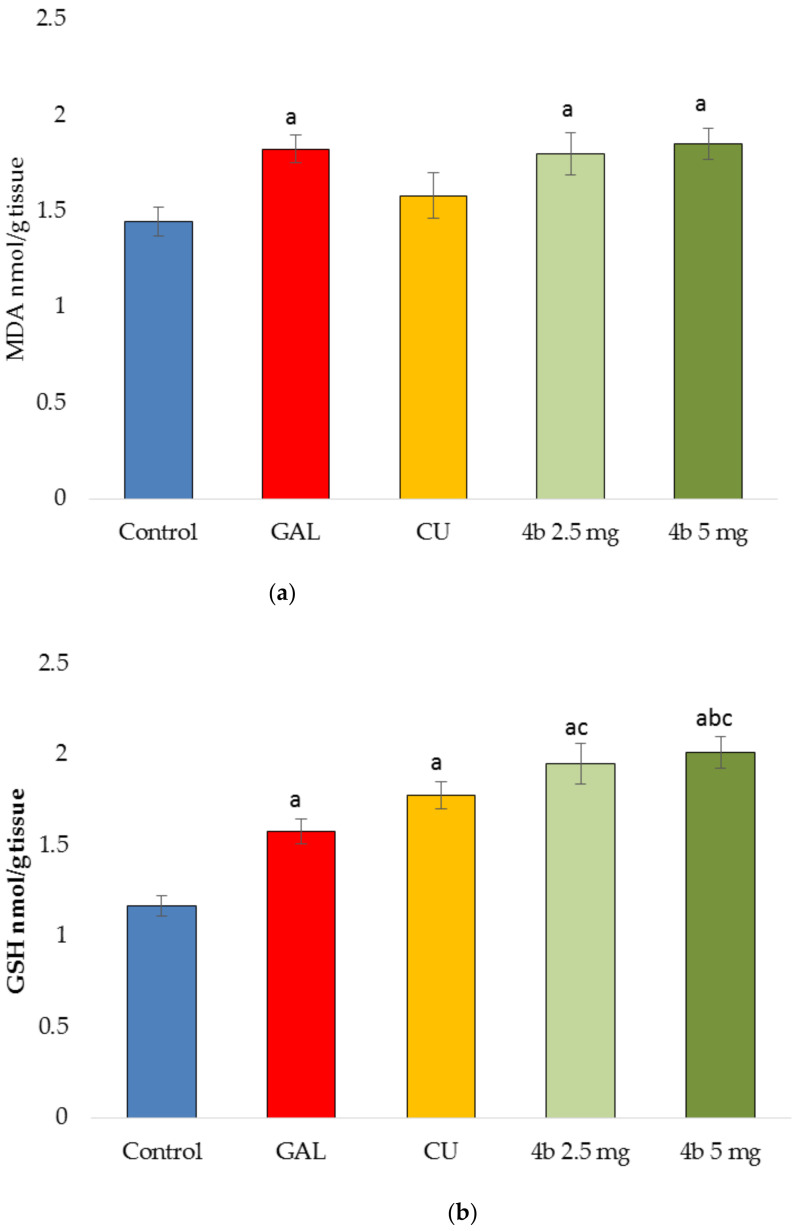
Antioxidant activity measured by malondialdehide (MDA) (**a**) and GSH levels (**b**) in brain homogenates derived from mice treated 14 days by GAL, CU, and **4b** in two doses (2.5 mg/kg and 5 mg/kg) or placebo (control group). ^a^*p* < 0.05 vs. control; ^b^*p* < 0.05 vs. CU; ^c^*p* < 0.05 vs. GAL.

The antioxidant activity of the tested compounds after 14-day treatment, was assessed by measuring the levels of malondialdehyde (MDA) and gluthathione (GSH) in mice brain homogenates as described in Materials and Methods. MDA is a specific marker for lipid peroxidation. The thiobarbituric acid (TBA) test is widely used to measure the formation of red pigment, which is an adduct of TBA with aldehydes, MDA, ketones and others. The effects of the tested compounds on MDA levels are given in Figure 3a. All compounds caused increase in MDA levels: CU—by 9%, GAL—by 26%, **4b** in dose 2.5 mg/kg—by 24% and **4b** in dose 5 mg/kg—by 28% compared to control. 

The oxidative stress causes decrease in GSH levels [9]. All tested compounds in the present study elevated the GSH levels (Figure 3b) showing antioxidant activity. The most prominent effect has **4b** in dose of 5 mg/kg (73%), followed by **4b** in dose of 2.5 mg/kg (67%), CU (52%) and GAL (35%). 

### 3.6. Hematological and Serum Biochemical Data

The hematological data after 14 days of treatment by GAL, CU, and two doses of **4b** or placebo are summarized in Table 1. There are no statistically significant deviations from the reference values for mice (embedded in the analyzer’s software), indicating no adverse effect of the test substances on hematopoiesis. 

The serum biochemical data collected after 14 days of treatment by GAL, CU, and **4b** in two doses or placebo are given in Table 2. No abnormalities on the hepatic or renal functions were observed apart from transaminases (ALAT and ASAT) and uric acid. An increase in both transaminases by **4b** was detected within the reference range. ALAT levels after treatment by the higher dose of **4b** are higher than the upper reference limit. The levels of uric acids were decreased by CU and **4b** relative to the control group but within the mice reference range. 

### 3.7. Antioxidant Activity Measured In Vitro

The antioxidant activity of GAL, CU and **4b** was assessed by two methods for radical scavenging (DPPH and ABTS) and one method for reducing power (FRAP) (Table 3). DPPH activity ranged from 4.63 μM TE/mM for GAL to 10.03 μM TE/mM for CU. **4b** showed an intermediate activity with 7.34 μM TE/mM. In the ABTS assay, **4b** demonstrated the highest capacity (101.22 μM TE/mM), stronger even than that of the positive control BHT (94.83 μM TE/mM). Regarding the reducing power, CU showed the highest activity with 8.70 μM TE/mM in FRAP assay followed by **4b** with 7.31 μM TE/mM and GAL with 5.69 μM TE/mM.

**Figure 4:**
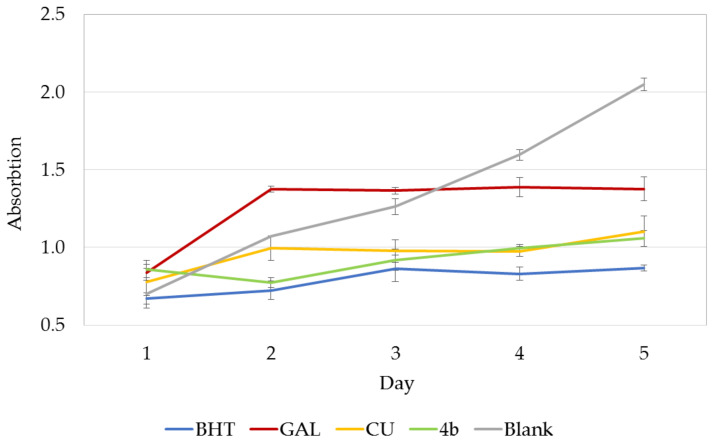
LPO inhibitory activity of GAL, CU and **4b** monitored for 5 days. Absorbance is given in mean ± standard deviation (*n* = 3). BHT is used as a positive control, MeOH as a blank.

The inhibition of lipid peroxidation (LPO) measured in linoleic acid system using FTC method was monitored for 5 days. The increase in absorbance indicates peroxide formation. Such an increase was observed initially for GAL and CU on day 2 but then the absorbance remained constant (Figure 4). A slight second increase was observed for CU on day 5. On the contrary, **4b** showed immediate decrease in absorbance on day 2 and then a gradual increase to the level of CU on day 4. On day 5, **4b** has a lower absorbance than both GAL and CU. Thus, **4b** protects better against peroxide formation than its leads GAL and CU. The in vitro measured LPO inhibition is in a good agreement with the MDA activity observed in mice brain homogenates. 

## 4. Discussion

In the present study, we performed in vivo and ex vivo studies of a newly synthesized hybrid between GAL and CU aiming to combine the AChE inhibitory properties of GAL with the powerful antioxidant properties of CU. Both properties have a beneficial effect on the delay of neurodegeneration. As a result, an AChE inhibitor was generated, 186 times more potent than GAL in vitro (IC_50_ of compound **4b** = 20 nM vs. IC_50_ of GAL = 3.52 μM) [19].

The LD_50_ of **4b** registered in the acute toxicity test on mice is 49 mg/kg orally. The LD_50_ of GAL in mice is 10 mg/kg i.p. [30] and between 15 and 45 mg/kg (median 30 mg/kg) orally [40]. The oral LD_50_ of CU is more than 2000 mg/kg (at highest given dose 2000 mg/kg) [41,42]. Obviously, the presence of CU fragment in the molecule of **4b** reduces its overall toxicity. The symptoms of toxic damage that were observed, expressed in breathing difficulty, initially rapid and then severely delayed, ataxia, lack of coordinated movements, tremor and tonic-clonic seizures, are characteristic of acetylcholine (ACh) neurotoxicity due to inhibition of AChE. In mammals, respiratory failure caused by inhibition of cerebral AChE is recognized as a cause of death [43]. Some authors have found that 50% of AChE inhibition may indicate intoxication or poisoning [44]. No changes in the morphology of the internal organs (lungs, liver, heart, kidneys, stomach, spleen, intestine, gonads and brain) were observed. The symptoms of cerebral AChE inhibition indicate that **4b** is able to cross the BBB and are in accordance with the in vitro measured permeability coefficient -log*P_e_* of 4.593. It should be noted that PAMPA assay measures the ability of compounds to cross the BBB only by passive diffusion. GAL is able to cross BBB partly by passive diffusion [45] and partly by the mediation of the choline transport system [46]. Whether **4b** as GAL derivative is able to use the same active transport system remains to be determined.

The daily administration of **4b** in doses of 2.5 mg/kg (1/20 of LD_50_) and 5 mg/kg (1/10 of LD_50_) for 14 days did not show any sign of toxicity in animals’ behavior. The ex vivo AChE activity measured after this period showed that **4b** inhibits the enzyme with 21% by the dose of 2.5 mg/kg and with 25% by the dose of 5 mg/kg compared to the control group (Figure 2). Such an extent of AChE inhibition is not enough to cause toxic effects or mortality, but it leads to a moderate increase in the levels of brain ACh. Any elevations of ACh levels in the brain are associated with improved cognitive effects [47], which was the purpose of the design of GAL-CU hybrids. The protective effect of GAL on mild to moderate Alzheimer’s disease is due to its inhibitory effect on AChE, which was confirmed in the present study by the 16% reduction in AChE activity in mice brain homogenates. The neuroprotective and AChE inhibitory effects of CU and other curcuminoids is well known [48,49,50]. Our study also confirmed these effects: CU showed 12% reduction in the AChE activity in the tested brain homogenates. Being a congener of GAL and CU, **4b** accumulates and fortifies these effects and exerts more powerful AChE inhibition than both leads. 

Tested in vitro, **4b** showed 186 times higher inhibitory effect on AChE than GAL and lower neurotoxicity than both GAL and CU [19]. In the present ex vivo studies, the difference in the reductions of AChE activity by **4b** and GAL is only 5–9% in healthy animals. Some authors report reactivation of AChE, or even overproduction of an enzyme in response to inhibition [51]. The mild difference in the ex vivo AChE inhibition between **4b** and GAL might be indicative for such recovery of AChE levels due to high spontaneous reactivation of the enzyme associated with rapid synthesis and release of a new enzyme from the liver. This possible mechanism requires further investigation. 

The AChE inhibition in vivo is associated with increased oxidative stress [52,53]. In response, the increased oxidative stress mediates an increase in the AChE activity. The association between AChE inhibition and increased oxidative stress also was observed in our MDA test. MDA is a biomarker of the oxidative stress relating to the lipid peroxidation. In the present study, we found that all tested AChE inhibitors elevated the MDA levels (Figure 3a). The stronger the AChE inhibition, the bigger the increase in MDA levels. At the same time, it was found in our GSH tests that all tested compounds increased the GSH levels (Figure 3b). GSH is an important endogenous cellular protector and antioxidant. The increased GSH levels might be interpreted as a compensatory response of the brain to overcome the lipid peroxidation induced by the decreased AChE activity. Kaur and Sandhir [54] conducted acute and chronic toxicity studies in Wistar rats with two different oral doses of insecticide (AChE inhibitor). In their study, rats showed a decrease in GSH levels after acute pesticide exposure and an increase in GSH levels during chronic repeated administration. The authors suggest that GSH elevation is an adaptive response to increased oxidative stress.

GAL has an antioxidant activity related with the enolic OH group [6]. One of the mechanisms by which GAL improves the mental function in Alzheimer’s dementia is thought to be its antioxidant effect [55,56,57]. CU also is a proven antioxidant and brain protector [58,59,60]. These effects were also confirmed in the present study, where CU and GAL significantly increased GSH levels by 52% and 35%, respectively, compared to the control group. As a hybrid of GAL and CU, **4b** accrues and augments the antioxidant effects of its leads. 

The in vitro antioxidant assays (DPPH, ABTS, FRAP and LPO inhibition) confirmed the results found ex vivo. CU has a powerful antioxidant activity which arises from the phenolic and/or enolic OH groups. The GAL enolic OH is preserved in **4b** in addition to the enone group in the linker. The hybrid **4b** showed DPPH radical scavenging and reducing properties higher than these of GAL and close to those of CU. The profile of inhibition of the lipid peroxidation of **4b** is also closer to CU than to GAL. However, an extraordinarily high activity of **4b** was detected in the ABTS assay. It can be speculated that this high activity is due to the conjugated enone fragment in **4b**, which is easily attackable by electron-rich species towards 1,4-addition. In comparison, CU exists mostly in enol form, which disturbs the α,β-unsaturated carbonyl system. 

The analyses of the hematological and serum biochemical data point to some concern about the elevated values of ASAT and ALAT by **4b**. ASAT levels are slightly higher than the upper limit but ALAT levels rise by 20% above the upper limit of the mice reference range. CU and **4b** decrease the serum levels of uric acid. CU inhibits xanthine oxidase and increases uricosuric activity [61,62]. As a CU congener, **4b** might be also involved in the same mechanism.

## 5. Conclusions

The current report is an initial preclinical study on a newly designed hybrid of GAL and CU, named **4b**, as a multitarget drug candidate for treatment of neurodegenerative disorders. The in vivo acute and short-term toxicity tests showed that **4b** is a low-toxic compound with an LD_50_ of 49 mg/kg administered orally in mice. Its strong AChE inhibitory activity in vitro, reported previously, was confirmed in the ex vivo test described here. As a hybrid of two antioxidants, **4b** showed expectantly an antioxidant activity measured by ex vivo MDA and GSH tests. Comparing to GAL and CU, **4b** exhibited antioxidant activity, higher than its leads. In the in vitro antioxidant tests, **4b** performed as a strong free radical scavenger, reducing agent and durable inhibitor of LPO. Thus, **4b** combines the strong anti-AChE activity of GAL with the durable antioxidant properties of CU and is a promising multitarget agent for treatment of neurodegenerative disorders. 

## Figures and Tables

**Table 1:** Hematological data after 14 days of treatment by GAL, CU, and two doses of **4b** or placebo. Values are means ± standard deviation (*n* = 6). Mice reference ranges are given according to analyzer’s software.

Complete Blood Count	Control	GAL	CU	4b 2.5 mg/kg	4b 5 mg/kg	Mice Reference Range
WBC × 10^9^/L	4.8 ± 0.62	3.9 ± 0.43	2.9 ± 0.22	5.4 ± 0.37	4.1 ± 0.2	2.9–15.3
LYM × 10^9^/L	3.4 ± 0.56	2.6 ± 0.72	2.6 ± 0.66	3.9 ± 0.5	3.0 ± 0.7	2.6–13.5
MO × 10^9^/L	0.7 ± 0.04	0.7 ± 0.05	0.5 ± 0.08	0.8 ± 0.04	0.6 ± 0.03	up to 0.8
GRA × 10^9^/L	0.6 ± 0.03	0.5 ± 0.04	0.4 ± 0.02	0.5 ± 0.03	0.5 ± 0.03	0.4–3.2
LYM %	71.6 ± 2.3	67.7 ± 2.8	68.9 ± 3.2	73.1 ± 3.6	70.1 ± 2.6	63.7–90.1
MID % (Mo + Eo + Ba)	15.5 ± 1.2	16.9 ± 1.4	14.2 ± 1.6	16.2 ± 1.3	17.4 ± 1.6	10–20
GRA %	12.9 ± 1.6	15.4 ± 1.8	16.9 ± 1.2	10.7 ± 1.4	12.7 ± 1.8	7.3–30.1
RBC × 10^12^/L	7.79 ± 0.3	7.45 ± 0.3	7.14 ± 0.2	7.77± 0.26	7.42 ±0.13	5.6–7.89
Hgb g/L	144 ± 7.8	139 ± 8.2	147 ± 5.4	150 ± 4.8	138 ± 5.8	120–150
HCT %	40.4 ± 1.8	37.7 ± 2.1	42.2 ± 1.6	40.2 ± 1.2	42.2 ± 2.2	36–46
MCV fl	53.2 ± 1.2	54.6 ± 0.9	53.8 ± 1.1	53.8 ± 1.4	63.8 ± 3.4	53.0–68.8
MCH pg	19.5 ± 1.3	18.6 ± 1.2	19.2 ± 1.2	18.9 ± 0.9	17.6 ± 1	16.0–23.1
MCHC g/L	341 ± 5.4	338 ± 5.8	332 ± 4.2	323 ± 3.8	340 ± 2.2	300–341
RDW %	14.7 ± 1.1	13.6 ± 1.2	12.6 ± 1.4	12.4 ± 1.1	11.4 ± 2.1	11.0–15.5
PLT 10^9^/L	736 ± 159	589 ± 143	430 ± 136	538 ± 151	632 ± 161	100–1610
PCT %	0.256 ± 0.06	0.272 ± 0.07	0.258 ± 0.1	0.267 ± 0.04	0.233 ± 0.06	0.1–0.48
MPV fl	6.2 ± 1.2	6.2 ± 1.6	6.0 ± 1.0	6.1 ± 1.4	6.0 ± 0.4	3.8–6.2
PDW fl	7.7 ± 1.5	9.2 ± 1.6	7.8 ± 1.2	8.3 ± 1.1	7.3 ± 1.6	7–23

Abbreviations: WBC, white blood cell count; LYM, lymphocytes; MO, monocytes; GRA, granulocytes; MID cells include less frequently occurring and rare cells correlating to monocytes (Mo), eosinophils (Eo), basophils (Ba); RBC, red blood cell count; HGB, hemoglobin; HCT, hematocrit; MCV, mean corpuscular or cell volume; MCH, mean corpuscular or cell hemoglobin; MCHC, mean corpuscular or cell hemoglobin concentration; RDW, Red cell distribution width; PLT, platelet count; PCT, platelet count test, MPV, mean platelet volume; PDW, platelet distribution width.

**Table 2:** Serum biochemical data after 14 days of treatment by GAL, CU, two doses of **4b** or placebo. Values are means ± standard deviation (*n* = 6). Mice reference ranges are given according to Refs. [38,39] and the reference values of our laboratory.

Serum Biochemical Data	Control	GAL	CU	4b 2.5 mg/kg	4b 5 mg/kg	Mice Reference Range
GLU mmol/L	6.2 ± 0.82	6.1 ± 0.77	5.,3 ± 0.76	6.1 ± 0.84	6.0 ± 0.36	4.2–7.5
UREA mmol/L	5.1 ± 0.32	4.0 ± 0.36	3.8 ± 0.28	4.5 ± 0.22	4.2 ± 0.32	3.27–12.1
CREAT µmol/L	68 ± 2.3	62 ± 28.8	65 ± 6.6	46 ± 5.6	56 ± 4.6	35–120
TP g/L	58 ± 2.2	53 ± 3.1	54 ± 2.6	53 ± 3.6	54 ± 2.6	53–63
ALB g/L	27 ± 1.8	26 ± 1.7	26 ± 2.2	26 ± 3.1	27 ± 2.3	26–29
ASAT U/L	83 ± 4.5	96 ± 5.2	102 ± 3.6*	123 ± 4.1 **	123 ± 5.3 **	65–122
ALAT U/L	58 ± 2.2	56.6 ± 3.1	62.2 ± 3.3	81.4 ± 3.4 **	96 ± 4.7 **	55–80
T-Bil µmol/L	5.6 ± 0.42	6.0 ± 0.38	5.6 ± 0.44	6.3 ± 0.28	7.2 ± 0.18	3.9–9.6
D-Bil µmol/L	2.4 ± 0.32	2.5 ± 0.44	2.4 ± 0.46	2.9 ± 0.34	3.6 ± 0.24	0–6.8
Ca mmol/L	2.04 ± 0.11	2.14 ± 0.15	2.04 ± 0.21	2.1 ± 0.2	2.2 ± 0.1	2.0–2.8
Uric acid µmol/L	195 ± 18.4	196 ± 20.3	133 ± 23.7 *	94 ± 16.7 *	104 ± 26.7 *	0–300
CHOL mmol/L	2.3 ± 0.35	2.61 ± 0.28	2.6 ± 0.44	2.16 ± 0.36	2.11 ± 0.23	1.77–2.9
TRIG mmol/L	1.04 ± 0.12	1.29 ± 0.18	1.02 ± 0.2	1.14 ± 0.22	1.04 ± 0.32	0.85–2.23
ALP U/L	148 ± 12.9	131 ± 13.6	122 ± 14.9	113 ± 13.8 *	123 ± 11.6	80–168
GGT U/L	13 ± 2.1	10 ± 2.6	10 ± 1.8	6 ± 3.2 *	8 ± 4.4	6–14
AMYL U/L	1021 ± 86.2	1179 ± 74.6	1036 ± 64.3	1263 ± 66.7 *	1068 ± 53.4	512–1312

*p < 0.05 vs. control; **p < 0.05 vs. reference values. Abbreviations: GLU, glucose; CREAT, creatinine; TP, total protein; ALB, albumin; ASAT, aspartate aminotransferase, ALAT, alanine aminotransferase; T-Bil, total bilirubin; D-Bil, direct bilirubin; Ca, calcium; CHOL, cholesterol; TRIG, triglycerides, ALP, alkaline phosphatase; GGT, gamma-glutamyl transferase, AMYL, amylase.

**Table 3:** Antioxidant activity (by DPPH, ABTS and FRAP assays) for GAL, CU and **4b**. Values are given in μM TE/mM ± standard deviation (*n* = 3). Butylhydroxy toluol (BHT) was used as a positive control.

Assay	GAL	CU	4b	BHT
DPPH	4.63 ± 0.08	10.03 ± 0.17	7.34 ± 0.43	46.37 ± 0.67
ABTS	5.87 ± 0.06	17.71 ± 0.58	101.22 ± 0.28	94.83 ± 0.26
FRAP	5.69 ± 0.46	8.70 ± 0.33	7.31 ± 0.32	10.55 ± 1.64

## Data Availability

Not applicable.

## Sample Availability

Samples of the compound are available from the authors.
